# Telehealth Care Through Internet Hospitals in China: Qualitative Interview Study of Physicians’ Views on Access, Expectations, and Communication

**DOI:** 10.2196/47523

**Published:** 2024-03-29

**Authors:** Yuqiong Zhong, Jessica Hahne, Xiaomin Wang, Xuxi Wang, Ying Wu, Xin Zhang, Xing Liu

**Affiliations:** 1 School of Humanities Central South University Changsha China; 2 Xiangya Hospital Central South University Changsha China; 3 Department of Psychological & Brain Sciences Washington University in St Louis St Louis, MO United States; 4 Center for Clinical Pharmacology The Third Xiangya Hospital of Central South University Changsha China; 5 Center for Medical Ethics Central South University Changsha China; 6 Medical Humanities Research Center Central South University Changsha China; 7 Office of International Cooperation and Exchanges Xiangya Hospital, Central South University Changsha China

**Keywords:** China, internet hospital, health care access, telehealth, doctor-patient relationship, mobile phone

## Abstract

**Background:**

Internet hospitals in China are an emerging medical service model similar to other telehealth models used worldwide. Internet hospitals are currently in a stage of rapid development, giving rise to a series of new opportunities and challenges for patient care. Little research has examined the views of chronic disease physicians regarding internet hospitals in China.

**Objective:**

We aimed to explore the experience and views of chronic disease physicians at 3 tertiary hospitals in Changsha, China, regarding opportunities and challenges in internet hospital care.

**Methods:**

We conducted semistructured qualitative interviews with physicians (n=26) who had experience working in internet hospitals affiliated with chronic disease departments in 3 tertiary hospitals in Changsha, Hunan province, south central China. Interviews were transcribed verbatim and analyzed by content analysis using NVivo software (version 11; Lumivero).

**Results:**

Physicians emphasized that internet hospitals expand opportunities to conduct follow-up care and health education for patients with chronic illnesses. However, physicians described disparities in access for particular groups of patients, such as patients who are older, patients with lower education levels, patients with limited internet or technology access, and rural patients. Physicians also perceived a gap between patients’ expectations and the reality of limitations regarding both physicians’ availability and the scope of services offered by internet hospitals, which raised challenges for doctor-patient boundaries and trust. Physicians noted challenges in doctor-patient communication related to comprehension and informed consent in internet hospital care.

**Conclusions:**

This study explored the experience and views of physicians in 3 tertiary hospitals in Changsha, China, regarding access to care, patients’ expectations versus the reality of services, and doctor-patient communication in internet hospital care. Findings from this study highlight the need for physician training in telehealth communication skills, legislation regulating informed consent in telehealth care, public education clarifying the scope of internet hospital services, and design of internet hospitals that is informed by the needs of patient groups with barriers to access, such as older adults.

## Introduction

As information technology develops rapidly in the current era, telehealth is growing exponentially in use [1-3]. Particularly in the wake of the COVID-19 pandemic, the use of telehealth for both primary and specialist care has accelerated around the globe [4]. In particular, telehealth is being implemented at an increasing scale in various countries with aging populations to improve health care access and quality for growing numbers of patients with chronic diseases [5-9].

The internet hospital is 1 major emerging telehealth model that is distinct to China, a country with a particularly large aging population and a high chronic disease burden [10-12]. Designed to make health care services more available, convenient, affordable, and efficient, internet hospitals are a type of online platform through which certain health care services can be conducted remotely. There are 3 main types of internet hospitals—those initiated by physical hospitals, those jointly established by physical hospitals and business enterprises, and those initiated by business enterprises relying on physical medical institutions. Research suggests that internet hospitals initiated solely by physical hospitals are the most widespread type [13]. In terms of the target patient population, internet hospitals primarily aim to facilitate services for patients with common illnesses requiring relatively simple treatment [14], patients with chronic diseases (diabetes, hypertension, and cancer) [15], and patients in remote and rural areas [16].

However, the scope of internet hospitals goes beyond telemedicine services for patients. Services provided by internet hospitals can be classified into three categories, (1) “core medical services,” which mainly include follow-up care for in-person medical services, telemedicine consultations, guidance on chronic disease management, and guidance on medication use; (2) “non-core medical services,” which mainly include medical consultations between health care providers and remote education for health care providers; and (3) “convenience services,” which mainly include health care appointment scheduling, mobile payment for health services, remote examination of medical test results, and dispensation and distribution of some medications [10,13,17,18]. Thus, the internet hospital model has the potential to increase access to health care for patients and training for providers, and to decrease costs across the health care system.

A number of recent policies by the Chinese national government have promoted rapid development and uptake of the internet hospital model. In 2015 [19] and 2018 [20], the State Council issued guidelines promoting “‘Internet +’ Healthcare,” which emphasized the development of internet hospitals as part of the “Health China” strategy for health care reform. Concurrently in 2018, the National Health Commission formulated specific regulations on internet hospital management, which officially authorized internet hospitals to facilitate a range of telehealth services and marked the start of their standardized development [21-23]. In 2020, the National Health Commission issued the “Notice on Strengthening Informatization to Support the Prevention and Control of the Novel Coronavirus Pneumonia Epidemic,” emphasizing the advantages of internet hospitals in controlling the spread of the COVID-19 pandemic [24]. In the wake of these policies, by June 2023, the number of internet hospitals had reached more than 3000, and 364 million of China’s 1.079 billion internet users were using online medical services [25,26]. However, research suggests that most internet hospitals are not yet fully developed or providing the full scope of services intended to achieve these goals [27-29].

At this early stage of the model’s development, little research to date has evaluated the views of Chinese medical professionals and patients regarding internet hospitals. However, research on telehealth in other countries reveals that telehealth raises many new concerns and challenges alongside the aforementioned opportunities [30-32]. One of the most common concerns raised by patients is the potential for misdiagnosis due to the inability to conduct physical examinations through telehealth [33,34]. Particular groups such as lower-income older adults also commonly report barriers to the use of telehealth such as lack of familiarity with technology or limited access to technological devices or internet connections [35]. Smartphone data or internet connection problems can also lower patient satisfaction and limit access among rural patient populations [36,37]. Various groups of patients also commonly report feeling concerned about patient privacy and the security and protection of medical data when using telehealth [38].

In order to guide the direction of internet hospital development in China, further research is needed to examine the emerging challenges and opportunities to patient care presented by this country-specific telehealth platform. The aim of this study was to explore the experience and views of chronic disease physicians at 3 tertiary hospitals in Changsha, China, regarding opportunities and challenges presented by internet hospital care.

## Methods

### Overview

The methodology whereby this study was designed and conducted is reported following the items in the Consolidated Criteria for Reporting Qualitative Research (COREQ) checklist [39]. See Multimedia Appendix 1 [39] for more information.

### Setting, Participant Recruitment, and Eligibility Criteria

We conducted in-depth, semistructured interviews with physicians at 3 tertiary hospitals in Changsha, Hunan Province, south central China. Inclusion criteria for participants were 18 years of age or older, experience working in internet hospitals, and employment in a chronic disease department at one of the study hospitals. Our rationale for these inclusion criteria was to select doctors who had work experience relevant to the research questions. Enrollment occurred over a 2-month period from April to May 2022. Using a purposive sampling approach, we obtained a list of doctors who had previous experience working in internet hospitals. We then messaged or called the doctors on the list to briefly explain the primary and secondary objectives of the study, invited them to share their perspectives related to the study, and asked them to be available for interviews. Out of the 28 doctors contacted, 26 agreed to participate, while 2 physicians declined due to a lack of time. Those who responded positively to the invitation were subsequently contacted by the author, YZ, either via WeChat (Tencent Holdings Limited) or telephone to schedule an interview.

We recruited participants until reaching data saturation, at which point no new information about the meaning of codes or themes and the relationship between them continued to appear [40].

In order to allow spontaneous answers and mitigate bias, participants were given minimum information in advance about specific interview topics.

### Ethical Considerations

In April 2022, the research protocol was approved by the institutional review board of Xiangya Hospital, Central South University (#202204092). No prior relationships existed between study participants and members of the research team. Verbal informed consent was recorded via an audio recorder for each participant before participation. Participants were informed in advance that their interviews would be recorded, with the assurance that these recordings would be subject to encryption for security purposes, and they provided their verbal consent accordingly. All participants received a compensation of 200 RMB (1 CNY=US $0.15 on May 2022) for their participation, which was disbursed through a WeChat transfer. To protect the information of the interviewees, the interview data were deidentified in the process of transcription from audio recordings.

### Data Collection

The interview guide was collaboratively developed and then subjected to pilot-testing by the research team. Throughout the concurrent phases of data collection and analysis, the interview guide underwent iterative refinement in response to emerging insights and participant responses. This adaptive approach is considered vital to the robustness of qualitative research [41]. Revisions were implemented subsequent to discussions involving YZ, JH, and Xiaomin Wang, aiming to clarify, define, and critically examine emerging content from interviews as relevant to the research questions. All questions from the finalized interview guide are listed in Textbox 1.

**Semistructured interview guide.**

Q1: What are your views on internet hospitals?

Q2: Could you tell me about your experience working in internet hospitals?

Q3: What do you think are the biggest advantages of internet hospitals? Can you give some examples?

Q4: What do you think are the most troubling or difficult aspects of internet hospitals? Can you give some examples?

Q5: How do you inform the patients who come to the internet hospitals before treatment?

Q6: Do you think there are any differences between doctor-patient communication in internet hospitals and physical hospitals?

Q7: What impact do you think internet hospitals have on doctor-patient communication? Can you give some examples?

Q8: What training have you participated in regarding internet hospitals? What do you think of this training?

Q9: What do you think about the current status of the development of internet hospital laws and regulations in China? How could they be improved? What other aspects can promote the further development of internet hospitals?

Q10: Is there anything else you would like to add about doctor-patient communication in internet hospitals?

Data for this study were collected from April to May 2022. We carried out interviews through a combination of online and offline modalities, depending on each participant’s preference and availability. Online interviews were conducted remotely by video call, via the mobile app WeChat. Offline interviews were conducted in private rooms at the study hospitals. The interviews were conducted in Mandarin Chinese by the authors, YZ (a postgraduate student) and Xiaomin Wang (an associate professor). Both interviewers have received professional training in qualitative interviewing and had extensive experience conducting qualitative research prior to this study.

The research team discussed possible probes and follow-up questions before beginning interviews, and interviewers used them when necessary to draw out more information relevant to the main research question. Concurrently, a second interviewer assumed the role of an observer to ensure the standardization of interview methods and to mitigate potential biases.

Interviews were audio recorded, transcribed, and uploaded into qualitative data management NVivo software (version 11; Lumivero) on password-protected computers to facilitate the analysis. Field notes made by interviewers during the interview process were also stored on password-protected computers, to be used for reference by the research team during analysis. Interviews ranged from 20 to 50 minutes long. Transcripts were sent to participants upon request, but no corrections, comments, or notes were made to transcripts.

### Data Analysis

Analysis of the data was performed through conventional content analysis, using guidelines described by Hsieh and Shannon [42]. An advantage of conventional content analysis is that it avoids using preconceived categories, to generate codes inductively from the data. This modality is considered appropriate when current knowledge of the phenomenon being researched is limited [42].

Authors YZ and Xuxi Wang transcribed all interviews verbatim and reviewed transcripts several times to acquire a thorough understanding of the whole data set. They then read transcripts line-by-line and highlighted keywords and sentences from a set of initial transcripts, to generate primary codes that captured key concepts. Primary codes were repeatedly reviewed and revised through discussion among the authors and comparison across the transcripts. A finalized codebook including 17 codes and 61 subcodes was used to code all interviews, using NVivo software (version 11). Data saturation was reached after 26 interviews, once the research team determined by the consensus that we had interviewed a sufficiently varied sample of physicians from the 3 study hospitals, while also having obtained sufficiently content-rich data.

Following the coding of all transcripts, all coded segments of the interview data were translated into English by authors YZ and Xuxi Wang, native Mandarin speakers, and double-checked for accuracy by author JH, a native English speaker. Codes and subcodes were repeatedly reviewed and were grouped into clusters according to similarities and differences. Clusters of codes were then treated as subcategories and aggregated into the main categories that were representative of the key findings. These categories were repeatedly reviewed until fully developed, through a process of identifying and comparing exemplary excerpts for each code, category, and subcategory.

This process of analysis culminated in three main categories describing the experiences and views of chronic disease physicians regarding the opportunities and challenges presented by internet hospital care, (1) advancements and shortcomings in care access due to internet hospitals, (2) patients’ expectations versus limitations on doctors’ availability and the scope of services—implications for doctor-patient boundaries and trust, and (3) advantages and downsides of online communication for comprehension and informed consent. These main categories are shown in Textbox 2, alongside the subcategories from which they were aggregated, and further explained in the results narrative below.

**Main study findings.**

**Advancements and shortcomings in care access due to internet hospitals**

- Advancements
  Enhanced ability to conduct follow-up care for patients with chronic illness
  More efficient channels for health education
- Shortcomings
  Disparities in access (ie, for older adults, patients with lower education levels, patients with limited internet or technology access, rural patients)

**Patients’ expectations versus limitations on doctors’ availability and the scope of services**

- Patients’ expectations of doctors’ availability create unclear professional boundaries.
- Patients’ expectations of the service scope of internet hospitals affect doctor-patient trust.

**Advantages and downsides of online communication for comprehension and informed consent:**

- Advantages
  Doctors value having extra time to think carefully about replies to patients’ messages, compared to in-person communication.
- Downsides
  Internet hospitals’ restrictions on consultation times, procedures, and arbitrary rules or schedules can hinder effective patient communication.
  Doctors have concerns about the quality of online diagnoses and advice, as well as patient accuracy and comprehension, due to the limitations of online care.
  Doctors have concerns about the completeness and uniformity of clinical informed consent in internet hospitals.

## Results

### Description of Study Participants

The 26 participants came from 3 different affiliated hospitals with 10, 5, and 11 participants interviewed from each hospital, respectively. Participants ranged in age from 29 to 49 years, and all 26 participants had PhD degrees. Only 5 participants stated that they had received specific training for working in internet hospitals, and 1 of them stated that training included discussion of clinical ethics in internet hospital care. We interviewed doctors from several departments involved in care for patients with chronic disease—oncology, cardiovasology, hematology, endocrinology, gastroenterology, nephrology, and infection departments. Aggregated participant characteristics are presented in Table 1.

**Table 1:** Participant characteristics (N=26).

Characteristics			Participants, n (%)
**Gender**			
	Male	10 (38)	
	Female	16 (62)	
**Department^a^**			
	Oncology	6 (23)	
	Cardiovasology	6 (23)	
	Hematology	1 (4)	
	Endocrinology	2 (8)	
	Gastroenterology	3 (12)	
	Nephrology	5 (19)	
	Infection departments	3 (12)	
**Job status**			
	Resident physician	1 (4)	
	Attending physician	20 (77)	
	Deputy chief physician	4 (15)	
	Chief physician	1 (4)	
**Work experience (years)**			
	<5	2 (8)	
	5-10	13 (50)	
	>10	11 (42)	
**Internet hospital work experience (years)**			
	<1	7 (27)	
	1-3	12 (46)	
	≥4	7 (27)	
**Received training specific to internet hospital care**			
	Yes	5 (19)	
	No	21 (81)	
**Received clinical ethics training in internet hospital care**			
	Yes	1 (4)	
	No	25 (96)	

^a^Some percentages may not add up to 100 due to rounding.

### Advancements and Shortcomings in Care Access Due to Internet Hospitals: Follow-Up Care, Health Education, and Disparities

Most doctors stated that internet hospitals affiliated with their physical hospitals of employment were still in the early stages of development and that their internet hospital work experience mainly took place in enterprise-initiated internet hospitals. Doctors stated that internet hospitals initiated by physical hospitals were “not fully operational yet,” (Dr B) and “the consultation volume of patients is relatively small” (Dr C). They also suggested that there was currently a “lack of incentives” (Dr D) to work in internet hospitals initiated by physical hospitals, whereas enterprise-initiated internet hospitals offered “higher income” (Dr B) and a setting where “doctors set their own prices” (Dr C).

In both internet hospitals initiated by physical hospitals and enterprise-initiated internet hospitals, doctors stated that the majority of their work consisted of online consultation for common or easily diagnosable diseases, and follow-up services for patients with chronic diseases who had previously received care at physical hospitals, such as adjusting medications and ordering medical tests to be scheduled in person. Most doctors were motivated to work for internet hospitals particularly because of the opportunity to be part of expanding follow-up care for patients with chronic diseases.

Most of the patients who come to the internet hospitals are chronic disease patients, with conditions such as hypertension, diabetes, coronary heart disease, etc. These patients have been clearly diagnosed in our hospital, and some of them need to be guided or communicated with about what needs to be paid attention to in the process of home-based management. For example, patients' blood pressure might fluctuate, or they can consult online if they have any uncomfortable symptoms, which is quite common.

Dr X

Some doctors also believed based on experience that internet hospitals could serve as a more efficient channel to provide health education for patients, particularly for the management of chronic diseases.

We also feel that doctors in tertiary hospitals do not have much time to do health education with patients, but through the internet hospitals platforms, we can explain to patients the concept of health or a healthy way of life. For example, for a patient with heart failure, he isn’t expected to come back to the hospital again and again, because I have instilled him with an understanding of healthy lifestyle and diet, and the workload of the doctor will be reduced in the long run.

Dr C

Despite the ways in which doctors felt internet hospitals expanded access to care and services, they had also observed disparities in access to internet hospitals across several groups, including older adults and patients with lower levels of education or technological literacy: “Older patients may not use smartphones or might need assistance to do so from family members” (Dr E).

Relatively speaking, if the patients come to the internet hospital for consultation, the education level of these patients will be higher, otherwise they will not be able to fully communicate with their doctor.

Dr O

Most doctors mentioned that internet hospitals are especially suitable for patients with chronic diseases. Doctors also stated that while older adults are one of the most common groups of patients with chronic diseases, many older adults have difficulties in using or accessing internet hospitals (Dr D and Dr X). Some doctors mentioned that internet hospitals currently have limited connections and overlap with local health services in rural areas. They believed moving toward more connection with local services was an important goal—particularly because the demand at tertiary hospitals frequently outstrips resources (Dr P and Dr Q), and because many rural patients travel long distances to receive care at tertiary hospitals.

Even for follow-up visits for chronic diseases or common diseases, many patients will still go to tertiary hospitals. Instead, the patient can go to a qualified local hospital and send us the results of the test, and then [through internet hospital care,] we can advise the patient or tell him how to adjust the medication, or refer the patient to a tertiary hospital for testing. But at present, internet hospitals have not played a big enough role in these aspects.

Dr E

### Patients’ Expectations Versus Limitations on Doctors’ Availability and the Scope of Services: Implications for Doctor-Patient Boundaries and Trust

#### Patients’ Expectations of Doctors’ Availability Create Unclear Professional Boundaries

When asked about new challenges in patient care posed by internet hospitals, only 1 doctor mentioned risks related to patient privacy and data security.

The internet hospital platform where I am located requires patients to provide their name, gender, age, ID number, and other information, which can be seen on the doctor's portal, but as a doctor I will definitely not disclose the patient's private information but just give him diagnostic advice according to the necessary information provided by the patient.

Dr G

By contrast, many doctors expressed concerns about their own privacy. Some doctors shared stories from their work in physical hospitals of willingly sharing their personal WeChat with patients in case patients had questions after discharge (Dr D, Dr M, and Dr R). While some doctors did not seem to mind-bending this boundary with patients, others remembered negative experiences when patients had sent messages making demands of doctors’ time at all hours (Dr E, Dr M, Dr P, and Dr Z). They also recalled times when patients obtained the doctor’s personal contact information through their own means and contacted them after leaving the hospital without the doctor’s consent (Dr V and Dr Z). As a result, some doctors had positive views of internet hospitals because they can serve as a means for online communication with patients without requiring the doctor to disclose their own personal contact information.

I prefer to use the official platform to communicate with patients, rather than through private WeChat or phone, because I really don't want to receive phone calls or text messages from patients after I work. But if the phone does ring, I will take into account that he is an old patient of mine, and I will still answer it, because I am not sure if there is any emergency. But for patients on such online hospital platforms, I rarely give them my phone number and personal WeChat.

Dr P

Several doctors were also uncomfortable that they were required to post information about themselves when working on internet hospital platforms, such as their name, location, and credentials (Dr P and Dr X). Because the audience of patients in internet hospitals is wider, they worried that patients who were dissatisfied with care may have the ability to post negative information about them in public forums online, citing their personal information. As a result, doctors stated that they would be more cautious in diagnosis and giving advice when dealing with patients in internet hospitals with whom they were less familiar (Dr J, Dr O, and Dr V).

#### Patients’ Expectations of the Service Scope of Internet Hospitals Affect Doctor-Patient Trust

Another concern expressed by many doctors was that patients held unrealistic expectations of the scope of services that internet hospital doctors provide. Some doctors mentioned that some patients feel that just because they spend money in an internet hospital, they should be able to get all their problems solved at once or get immediate treatment (Dr E and Dr G)—when in reality in many cases, the doctor might need to conduct tests over multiple online consultations or might recommend that patients seek further medical services in offline, physical settings. Doctors were concerned that patients’ dissatisfaction with unmet expectations might generate distrust toward the doctor.

What patients don't know is that in fact, most of the time, seeing a doctor is a step-by-step process, and it is necessary to do examinations step-by-step to exclude diseases or diagnose diseases. They often have high expectations for the effect of consultation in internet hospitals, and they think that doctors should be able to diagnose their diseases at one time; and not only to diagnose them, but also to propose a treatment plan.

Dr E

Some doctors suggested that this gap between expectations and reality could be especially strong for new patients whom the doctor had not seen previously for in-person care. They described that they often recommend for new internet hospital patients to go to physical hospitals to be examined before receiving further internet hospital care or advice, and that patients who are expecting immediate solutions can find this disappointing (Dr U and Dr X). One doctor suggested the need to educate patients and the general public on the scope of internet hospital care, in light of this mismatch in patients’ and doctors’ expectations (Dr Y).

However, some doctors raised concerns about issues related to doctor-patient trust that had less to do with adjusting patients’ expectations and more to do with the format of online communication itself.

Doctor-patient trust will be better in physical hospitals. Because the doctor-patient relationship is a very special relationship, offline communication can observe the patient's expression, speed of speech, action, etc, and is more suitable for empathy with patients. When it comes to the doctor-patient relationship and trust, I think face-to-face consultation is still necessary.

Dr J

Face-to-face communication in physical hospitals may be more detailed, because if it is through text messages or phone calls, I may be able to talk to the patient in a few words, but if the patient is communicating in our hospital, it may take me half an hour. Because there is unequal information in medicine itself, the patient himself is not very clear about medicine, and without adequate communication, there is no trust between doctors and patients.

Dr W

### Advantages and Downsides of Online Communication for Comprehension and Informed Consent

Doctors working in internet hospitals mainly used pictures and texts, and rarely video calls, to communicate with patients. Some doctors valued the extra time gained by this format to think carefully about their replies to patients’ messages (Dr E, Dr U, and Dr X). However, most doctors pointed out how the lack of nonverbal communication could increase miscommunication and misunderstandings.

Online communication is through typing, and some doctors can't see the facial expressions of patients, which is very inconvenient. The communication between doctors and patients may need body language, facial expressions and other aspects.... I want the patient to really understand me in terms of attitude or tone or feeling or whatever.

Dr G

Doctors also expressed dissatisfaction with limitations on the time and procedures for consultation through various internet hospitals, and how sometimes arbitrary rules or schedules hindered communication with patients.

The doctors in our department need to be on duty every month in the internet hospitals. When it is my turn to be on duty, a patient will send his questions to me through the platform, but I think this mode is not good. For example, the patient might leave a message for me, but I am busy and don’t reply to him in time, and he may not see my reply in time when I reply. If I go back and forth with him several times, this problem will not be solved until I come back on duty next month, and then the patient's problem will not be solved at all.

Dr C

The internet hospital at our hospital stipulates that patients can ask five questions at a time.... Sometimes doctors are not able to inquire in detail in order to understand the condition.

Dr G

Doctors had concerns about the quality of diagnoses and advice that they provided online, due to the inability to do direct physical examination. These concerns were intensified by their perception that many patients could not describe their symptoms clearly and accurately.

Currently, a lot of people still lack of basic medical knowledge, which will lead to ineffective or inefficient consultation on the internet, because they cannot describe their own symptoms, or cannot collect their own data and then summarize it. Patients cannot provide information about their condition sufficiently and accurately, which will seriously affect the efficiency of consultation.

Dr E

Two doctors also specifically mentioned the difficulty in internet hospital care of not being able to use the “four-diagnosis method”—a method used by doctors in traditional Chinese medicine for diagnosing illness, including diagnosis through observation, diagnosis through auscultation and olfaction, diagnosis through inquiry, and diagnosis through pulse feeling [43]. Although doctors in this study practiced mainly “Western” medicine, they described integrating certain traditional practices such as this method into their care at physical hospitals (Dr D and Dr H).

In light of concerns about the potential for miscommunication with patients, a few doctors also expressed uncertainty about the completeness and uniformity of clinical informed consent as it is currently practiced in internet hospitals. While they believed that a standardized process of online informed consent for medical advice and treatment was needed, they did not know of any relevant laws or procedures.

Because we have so little time to communicate online, and such a narrow scope of care services, we don't usually obtain informed consent online. I might listen to the patient explain his symptoms. I might tell him what tests he needs before I give my advice, or if I'm dealing with a familiar patient, I might just prescribe his medication, so there's no need for informed consent. However, I think how to issue online informed consent, whether online informed consent is legally effective, and how to sign online informed consent all need to be considered. This is also for the protection of medical staff.

Dr U

## Discussion

### Principal Findings

This study sheds light on previously underresearched aspects of internet hospitals in China, as both the first interview study to examine physicians’ perceptions of internet hospitals and one of the few studies on internet hospitals conducted in China outside of its most major cities. Our research revealed that physicians see enhanced opportunities in internet hospitals to conduct follow-up care for patients with chronic illnesses and to provide health education. However, physicians noted disparities in access for different groups, such as older adults, patients with lower education levels, patients with limited internet or technology access, and rural patients. One particularly novel finding was the conflict between patients’ expectations and the reality of limitations on doctors’ availability and the scope of services available through internet hospitals. Physicians perceived that this gap affected both boundaries and trust in the doctor-patient relationship. Physicians also discussed opportunities and challenges in doctor-patient communication, including issues of comprehension and informed consent. Considering that the development of internet hospitals involves multiple industries, including medical institutions, national policymaking departments, and technology providers, we raise several suggestions below on physician training, patient education, regulations, and design, as well as directions for future research.

### Training for Doctors

Internet hospital care involves real-time online sharing of medical data. Information about both doctors and patients is centralized and easily accessible to authorized users on the internet hospital platform. Some doctors in our study were uncomfortable when required to publicly post their names, basic personal information, and credentials on internet hospital platforms because it might make them more vulnerable to public criticism. Doctors’ reasons for being concerned about this were in line with previous research showing a high degree of conflict in the doctor-patient relationship in China [44,45]. This underscores the importance of current efforts both locally and internationally that aim to rebuild trust in the doctor-patient relationship [46-48]. Considering doctors’ concerns about patients requesting for them to disclose their WeChat in both physical and internet hospital work, communication skills training for doctors should prepare doctors for how to interact with patients with empathy and care, while also maintaining their preferred professional boundaries.

It was also notable that only 1 doctor who was interviewed discussed concerns related to patient privacy and data security when asked about challenges presented by internet hospitals. By contrast, Li et al’s [49] study on the determinants of patients’ use of internet hospitals in China showed that while patients generally desire to use internet hospitals, they are apprehensive about the associated risk of their personal information being leaked. Due to the heightened potential for data leaks and breaches of patient health information associated with the use of internet hospitals, it is imperative that health care professionals undergo training to raise their awareness of data security precautions. For instance, physicians should be trained on proactive measures that they can take to guarantee that the internet hospital services they are affiliated with implement adequate security protocols around patient information. Furthermore, physicians should be trained to communicate with internet hospital patients or their legal proxies about potential risks related to data security and to apprise them of protective measures enacted to safeguard information. Future research should also evaluate the frequency with which data leaks and breaches in internet hospitals actually occur.

Findings from our study also suggest that internet hospitals have led to changes in doctor-patient communication. Doctors in our study considered it to be an advantage of internet hospital care that they generally had more time to communicate with patients compared to in-person care. However, a previous study on internet hospitals suggested that while doctors can obtain key information from patients within a few minutes through in-person communication and examination, information received in the same amount of time online tends to be more limited [43]. Research conducted by Deng et al [50] also highlights that engaging in online consultation work while simultaneously engaging in a main career providing in-person medical consultation may place excessive demands on doctors’ time and energy. This phenomenon of work overload could potentially impede the widespread adoption of internet hospitals and introduce added risk to medical practice.

Relatedly, doctors in our study mentioned that when working in internet hospitals, they could only communicate with patients in the form of text, pictures, or video-based consultations, and that they had to rely largely on patient self-report. Both of these factors caused doctors to worry about the accuracy of their diagnoses. This aligns with recent research showing that about 70% of surveyed health care providers believe communication difficulties between patients and health care providers result in online consultations being insufficient [51], and about 70% of providers report feeling apprehensive about the possibility of misdiagnosis when providing care through internet hospitals [51]. Recent research has also found that patients who use internet hospitals have more negative views on the doctor-patient relationship than nonusers—including both interpersonal factors such as the degree to which patients trust doctors and practical factors such as the degree to which patients agree with their doctors’ medical opinions. Studies from other countries have similarly shown that telehealth can present new challenges or deficiencies in communication [52-55].

To address such challenges, telehealth communication competencies need to become a core component of both future research and physician training for internet hospitals in China—just as similar competencies are emerging as a priority for telehealth enhancement around the world [56]. Physicians providing care through internet hospitals should undergo standardized training for web-based communication skills, as research from other countries suggests such training can adapt interpersonal skills to the telehealth environment [57] and enhance empathic expression. More training for physicians on this skill set might reduce their apprehension about communicating through internet hospitals and assist them in communicating in a manner that improves outcomes for patients. Considering that doctors in our study expressed concern about patient comprehension and diagnostic accuracy, further research is also needed to evaluate and establish methods for measuring patient satisfaction, patient comprehension of information communicated by doctors, and diagnostic accuracy in internet hospital care. Future research should also examine the feasibility of integrating traditional Chinese medical practices such as the four-diagnosis method into telehealth care in China.

### Education for Patients or the Public

Findings from our study highlight new challenges in the doctor-patient relationship posed by internet hospital care. One especially novel finding in our study was doctors’ perception that patients subconsciously expected them to be online 24 hours a day, while doctors actually had limited hours working in the internet hospital and could not meet this expectation. Particularly when patients still needed to ask questions after the end of the physician’s available time for consultation, doctors described the risk of conflicts with patients. These findings suggest many patients may be unaware when message-based interactions with physicians in internet hospitals are discontinuous or asynchronous. Therefore, public information about internet hospitals should specify the boundaries of physicians’ availability for internet hospital consultations. While the scope of services may expand as internet hospitals continue to develop, information disseminated to the public should make it clear that internet hospital care is currently only intended for either follow-up care for previously diagnosed patients with chronic diseases, or for new patients with common and more easily diagnosable conditions. Finally, public education should equip patients or their proxies for distinct ways in which they might self-advocate for optimal care in the context of internet hospitals compared to in-person care. This may involve the development of interventions such as question-prompt lists that are specific to equipping patients for internet hospital consultations.

### Regulations and Laws

Doctors in our study believed that difficulties with nonverbal communication in internet hospitals often led to miscommunication and misunderstanding, and many raised concerns that there were no specific laws regulating online doctor-patient communication. As a result, most doctors in our study expressed that they felt they were walking on eggshells concerning possible conflicts with patients. This builds on findings from the “2022 China E-hospital development research report” [51], in which one of the most common suggestions made by health care providers for the further development of internet hospitals was to standardize legal protection for doctors practicing in internet hospitals. Gaps in relevant laws and regulations may reduce the willingness of risk-conscious clinicians to provide medical services through internet hospitals.

Existing internet hospital laws and regulations in China are still mainly in the trial stage [51,58], and are being outpaced by evolving challenges in internet hospital care. The doctors in our study believed that internet hospitals may increase the difficulty of diagnosis and treatment, increase medical safety risks related to miscommunication, and increase the risk of medical malpractice liability. Research by Zhi et al describes how the inability of doctors to perform physical examinations or certain laboratory or imaging examinations through internet hospitals may compromise the accuracy of doctors’ judgments [59]. However, the legal responsibilities of physical medical institutions, internet hospitals, and doctors regarding issues such as these have not been fully clarified. We suggest that further refinement and clarification of these and other aspects of internet hospital law will help doctors feel more protected in their work and increase the motivation of doctors to work in internet hospitals.

Doctors in our study mentioned that China also lacks detailed legal provisions on the implementation of online informed consent. Internationally recognized ethics standards highlight 4 core elements of informed consent—capacity to consent, information disclosure, comprehension, and voluntary authorization [60]. Informed consent issues involved in telehealth in other countries are similar to those described by doctors working in internet hospitals in this study, namely, the degree of discernment required from providers to ensure that patients are sufficiently informed to provide consent increases dramatically in telehealth [61]. In the United States, different states have different regulations on remote informed consent, and no federal policy has been formed at present. Some states require patients to fill out and sign written consent forms, while others do not [62]. In China, the Administrative Measures for internet hospitals stipulate that “internet hospitals must warn patients of risks and obtain informed consent from patients” [22]. However, current laws in China do not provide clear rules regarding the validity of electronic signatures for informed consent in internet hospital care. Informed consent in internet hospital care also involves unique information security issues due to the use of electronic health records, but there is currently no specific legal guidance for internet hospital platform developers or doctors concerning data security protection of informed consent in internet hospital care.

### Tackling Disparities

Our research revealed that while older adults are at higher risk for chronic illness and are the main target population for internet hospitals, they are also reported by doctors to experience a number of barriers to internet hospital use. This finding aligns with research from various countries showing that older adults are less likely than younger patients to express positive attitudes toward using telehealth [63,64]. Health care providers in China and other countries have observed that older adults may be apprehensive about telehealth due to difficulty in operating computers or smartphones [65], may need the help of care partners to log into telehealth accounts successfully, and may need more time on average to download and set up applications [43,66]. Previous surveys have also shown that medical personnel believe that the low efficiency of online communication between doctors and patients and the low internet use rate of some patient groups (such as older adults) are the main factors hindering the development of internet hospitals [51].

Previous research in various countries has shown that the ease of use and perceived usefulness of telehealth systems have a positive impact on the acceptance of telehealth in patients who are older [67,68]. However, to date, China has not established an effective quality control system for internet hospitals [27,69], and the aforementioned ways in which internet hospitals currently pose increased risks for patient safety may affect general patients’, let alone older adults’ willingness to use them [49]. We recommend that the needs of older adults be considered in the design and development of internet hospital platforms and that older adults participate in the system design process [70,71]. Community health workers may be a workforce that could be mobilized to support telehealth training efforts among patients who are older, assist individuals with limited telehealth literacy in attending online appointments, and provide culturally and linguistically appropriate information about telehealth to rural patients and communities [64,72,73]. In general, health care organizations should invest in developing internet hospitals that are functional and easy to use. Drawing from research on telehealth design improvement in other countries, internet hospitals could be designed with features in mind to help physicians communicate more clearly with patients, such as providing notifications to physicians when patients read messages [74]. In addition, it may be beneficial for platforms to provide training materials to patients when patients register and log into internet hospitals for the first time [75]. Considering that a major goal of internet hospital development is to expand health care access, it will be crucial to address disparities in internet hospital use through these and other educational and design considerations.

### Limitations

This study should be interpreted in the light of certain limitations. As most participants interviewed were attending physicians, findings may not be generalizable to the perspective of other health care workers or patients. The generalizability of our study findings from a single region and time point may also be limited, as there may be variations in internet hospital features and practice in other regions in China, and over time as internet hospitals continue to develop rapidly.

### Conclusions

This study explored the experience and views of physicians in 3 tertiary hospitals in Changsha, China regarding access to care, patients’ expectations versus the reality of services, and doctor-patient communication in internet hospital care. Findings from this study indicate that there is a need to train physicians in telehealth-specific communication skills. National policymaking departments should also further refine laws and regulations concerning internet hospitals, particularly those related to online informed consent. Technology developers should take the needs of older adults into particular account in the design of internet hospital platforms.

## Appendix

Multimedia Appendix 1 COREQ (Consolidated Criteria for Reporting Qualitative Research) checklist.

## Abbreviations

COREQ: Consolidated Criteria for Reporting Qualitative Research

## Data Availability

The data generated and analyzed during this study are available from the corresponding author upon reasonable request.
